# A Bibliometric Review of Actigraphy-Based Sleep Research in Diabetes

**DOI:** 10.7759/cureus.82058

**Published:** 2025-04-11

**Authors:** Ayesha Juhi, Himel Mondal

**Affiliations:** 1 Physiology, All India Institute of Medical Sciences, Deoghar, IND

**Keywords:** actigraphy, bibliometric analysis, circadian rhythm, diabetes, glucose monitoring, insulin resistance, metabolic health, sleep, sleep disorders, wearable technology

## Abstract

Actigraphy is a valuable tool for objectively assessing sleep patterns, with growing interest in its application to sleep research in patients with diabetes mellitus. Sleep disturbances are increasingly recognized as modifiable risk factors for metabolic dysregulation, yet the research landscape in this domain remains unclear. This bibliometric analysis aimed to evaluate global research trends, influential contributors, and thematic developments in actigraphy, sleep, and diabetes. Data were retrieved from PubMed, covering the period from 2005 to 2024, and analyzed using Biblioshiny and VOSviewer to assess publication growth, collaboration networks, keyword co-occurrence, and thematic evolution. A total of 203 publications from 101 sources were identified, with an annual growth rate of 18.11%, indicating rising research interest. Contributions from 1,170 authors demonstrated strong collaboration, with 10.84% of studies involving international co-authorship. Core journals, including SLEEP, SLEEP MEDICINE, and JOURNAL OF SLEEP RESEARCH, played a dominant role, while institutions such as Kyoto University and the University of Illinois at Chicago emerged as leading research hubs. Thematic analysis revealed a transition from foundational studies on glucose metabolism and polysomnography to investigations of circadian rhythms, actigraphy-based sleep monitoring, and metabolic health. Recent trends highlight emerging topics such as gestational diabetes and mental health, signaling shifting research priorities. While collaboration networks indicate strong regional clusters, greater international partnerships are needed to improve research diversity and applicability. This study highlights the expanding role of actigraphy in sleep and diabetes research, with implications for adaptive medicine, artificial intelligence-driven sleep analytics, and digital health interventions.

## Introduction and background

Sleep disturbances are increasingly recognized as a modifiable risk factor in the development and management of diabetes [1]. Poor sleep quality, insufficient sleep duration, and circadian misalignment have been linked to glucose metabolism dysregulation, insulin resistance, and an increased risk of developing type 2 diabetes [2]. In individuals with diabetes, disrupted sleep patterns contribute to worsened glycemic control, increased cardiovascular complications, and poorer overall health outcomes [3]. While traditional sleep assessment methods such as self-reported questionnaires and polysomnography (PSG) have been widely used in sleep research, they have notable limitations [4]. Self-reports are subject to recall bias and lack objectivity [5], while PSG, though accurate, is resource-intensive, requires a clinical setting, and provides only short-term data [6]. These challenges underscore the need for accessible, real-world, and continuous sleep monitoring tools to better understand the role of sleep in diabetes. 

Actigraphy has emerged as a promising, non-invasive, and wearable technology that enables long-term, real-time monitoring of sleep-wake cycles and circadian rhythms [7]. Using wrist-worn accelerometers, actigraphy objectively measures parameters such as total sleep time, sleep efficiency, wake-after-sleep onset, and sleep fragmentation over extended periods [8]. This provides a comprehensive understanding of sleep patterns compared to single-night PSG studies. In diabetes research, actigraphy has been used to explore the impact of sleep duration, variability, and circadian misalignment on glucose homeostasis, insulin sensitivity, and diabetes complications [9]. Despite its growing application in sleep and metabolic health studies, no systematic bibliometric analysis has mapped its research trends, influential contributors, or evolving themes. This study aims to fill this gap. 

Bibliometric analysis is a quantitative method to evaluate research trends, authorship patterns, and influential publications using publication data. In health sciences, it helps identify emerging topics, research gaps, and leading contributors or institutions. This evidence-based mapping supports informed decision-making in policy, funding, and academic research planning [10]. By systematically mapping the existing literature, this study aimed to identify publication trends, influential authors, research hotspots, and collaboration networks related to actigraphy, sleep, and diabetes. Understanding these patterns will help researchers and clinicians recognize gaps in current knowledge, facilitate interdisciplinary collaboration, and guide future investigations into actigraphy’s role in diabetes management. 

## Review

Methods

This bibliometric study analyzed research trends, influential contributors, and thematic developments in actigraphy, sleep, and diabetes using PubMed as the sole database. PubMed is a well-established search portal that sources the data from three databases - Medline, PubMed Central, and Bookshelf - and provides access to high-quality, peer-reviewed publications relevant to this field [11]. To capture the evolution of research over time, the study will include publications from 2005 to 2024, covering nearly two decades of advancements. Publications in all languages will be included to ensure a comprehensive global perspective. As PubMed is the major database of biomedical literature, we used it as our data source. In addition, we did not have access to Web of Science, Scopus, or Embase databases.

A structured search strategy was employed using the query: (“actigraph”[tiab] OR “actigraphy”[tiab]) AND (“sleep”[tiab]) AND (“diabetes”[tiab]). All records were screened for duplicates and incomplete data, ensuring a refined dataset for analysis. However, there were no incomplete data, and all the studies were included in the final analysis. The extracted bibliographic information included titles, authors, affiliations, journals, keywords, countries, and other information. The data were exported in PubMed format for its easy acceptability in the analysis software we used in this study. 

For analysis and visualization, two key bibliometric tools were used. Biblioshiny, an R-based extension of the Bibliometrix package, was employed for descriptive statistics, trend mapping, and thematic evolution analysis [12]. To access this, we used RStudio 2024.12.1 Build 563 (Posit Software, PBC, Boston, MA). VOSviewer version 1.6.20 (Copyright 2009-2023 Nees Jan van Eck and Ludo Waltman; Leiden, Netherlands) was used to generate network visualizations, including co-authorship and keyword co-occurrence [13].

Since this study is based on publicly available bibliographic data from PubMed, no ethical approval is required.

Results

The bibliometric analysis identified 203 publications from 101 sources, with an 18.11% annual growth rate, reflecting the rising interest in this field. A total of 1,170 authors contributed, with 8.32 co-authors per paper, indicating strong collaboration. 10.84% of studies involved international co-authorship, and no single-authored papers were found. The research featured 720 unique keywords, with an average document age of 6.28 years. The findings highlight the increasing global focus on actigraphy’s role in sleep and diabetes, emphasizing its clinical relevance and research expansion.

Publication Trend

**Figure 1:**
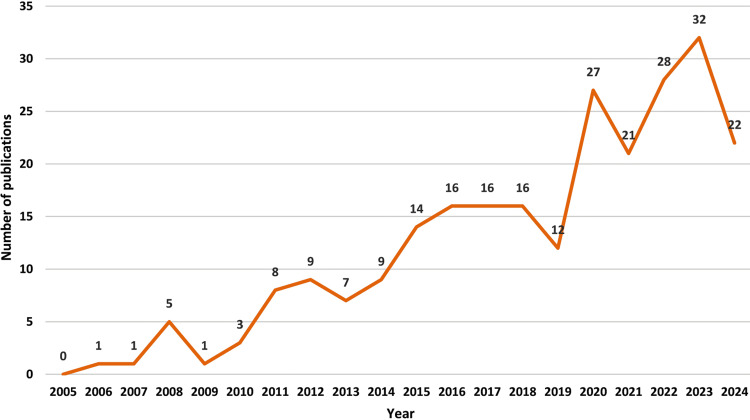
Number of publications from 2005 to 2024 Figure generated in Microsoft Excel with PubMed data by Dr. Himel Mondal.

The publication trend shows a steady rise in research on actigraphy, sleep, and diabetes over the years (Figure 1). 

The early years (2006-2012) saw a low but gradually increasing output. A significant upward trend is evident from 2014 onward, with sharp growth post-2019, peaking in recent years. The surge reflects the growing recognition of actigraphy’s role in sleep monitoring and metabolic health. Despite minor fluctuations, the overall trajectory indicates sustained and expanding interest in this interdisciplinary field.

**Figure 2:**
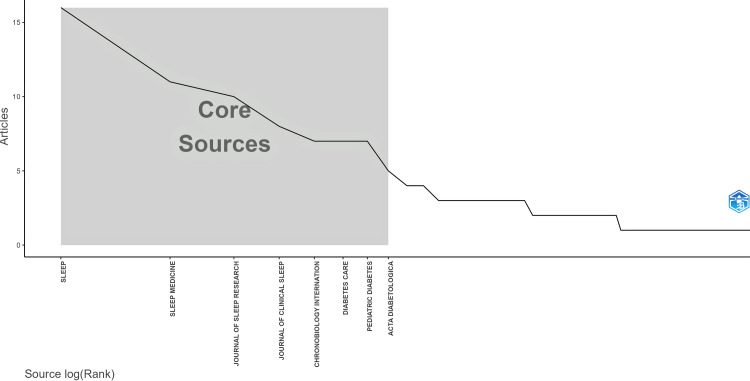
Core sources of the articles by Bradford’s law This figure is generated from Biblioshiny.

The core sources (i.e., journals) of the articles are shown in Figure 2 [14]. 

The concentration of research within core journals suggests that a few key outlets drive the discourse on actigraphy in sleep and diabetes, highlighting potential publication bias. In the provided visualization, journals like SLEEP, SLEEP MEDICINE, and JOURNAL OF SLEEP RESEARCH form the core sources, publishing the highest number of relevant articles. Beyond this core, the number of articles per journal declines sharply, with more specialized journals like Chronobiology International and Pediatric Diabetes contributing fewer publications. This pattern reflects the typical distribution in scientific literature, where key journals dominate while numerous others provide occasional contributions.

Authorship and Affiliations

**Figure 3:**
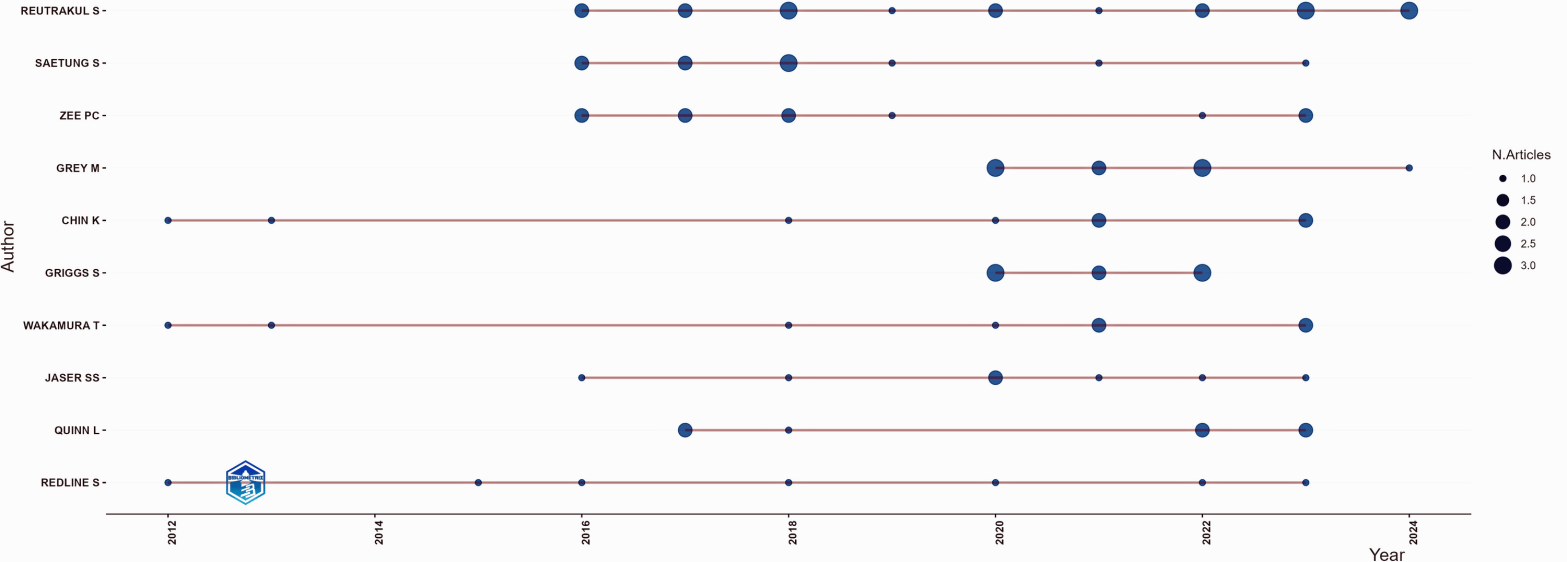
Publications of top authors according to years This figure is generated from Biblioshiny.

Figure 3 illustrates the publication trends of various authors over time, showcasing their research output and impact. 

The horizontal lines represent the active publishing periods for each author, while the size of the blue circles indicates the number of articles published in a given year. Authors such as Reutrakul S, Saetung S, and Zee PC demonstrated a consistent and high research output in recent years, whereas others have sporadic contributions. This visualization highlights the concentration of research activity among key contributors while indicating varying degrees of sustained academic engagement.

**Figure 4:**
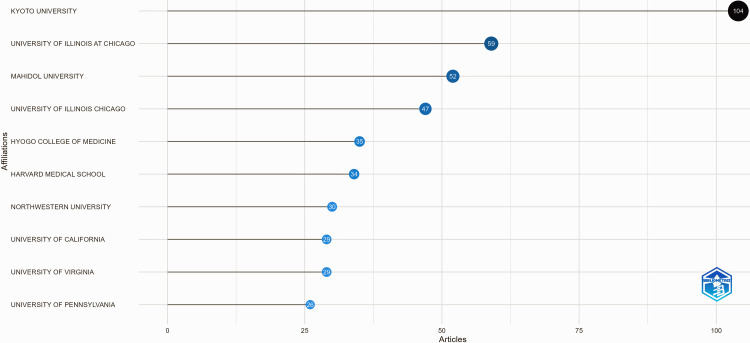
Most relevant affiliations in the research domain This figure is generated from Biblioshiny.

Figure 4 illustrates the most relevant affiliations contributing to the research field based on the number of published articles. 

Kyoto University leads with the highest number of publications (104), followed by the University of Illinois at Chicago (59) and Mahidol University (52). Other institutions, including the University of Illinois Chicago (47), Hyogo College of Medicine (35), and Harvard Medical School (34), also have a significant presence. The distribution highlights the prominent role of academic and medical institutions in advancing research within this domain. The varying sizes of the data points further emphasize the differences in research output among these affiliations.

**Figure 5:**
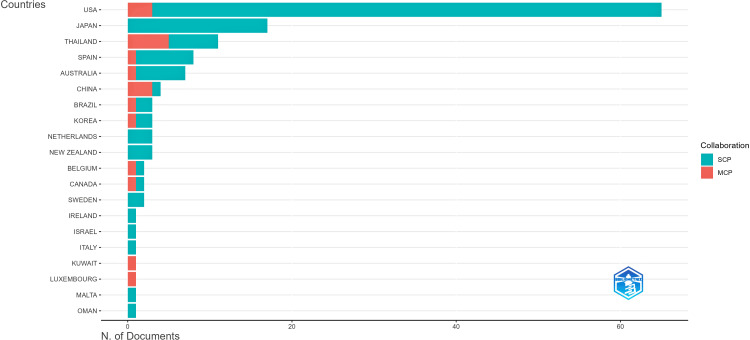
Corresponding authors' countries in the research domain This figure is generated from Biblioshiny. SCP: single-country publications; MCP: multiple-country publications.

Figure 5 presents the distribution of corresponding authors' countries based on the number of documents published. 

The United States leads with the highest number of publications, followed by Japan and Thailand. Spain, Australia, and China also contribute significantly to the research field. The chart differentiates between single-country publications (SCP) and multiple-country publications (MCP), highlighting the extent of international collaboration. The predominance of SCP suggests that much of the research is conducted within individual countries, although MCP segments indicate cross-border collaborations, particularly in countries like Thailand and China.

Keywords and Themes

**Figure 6:**
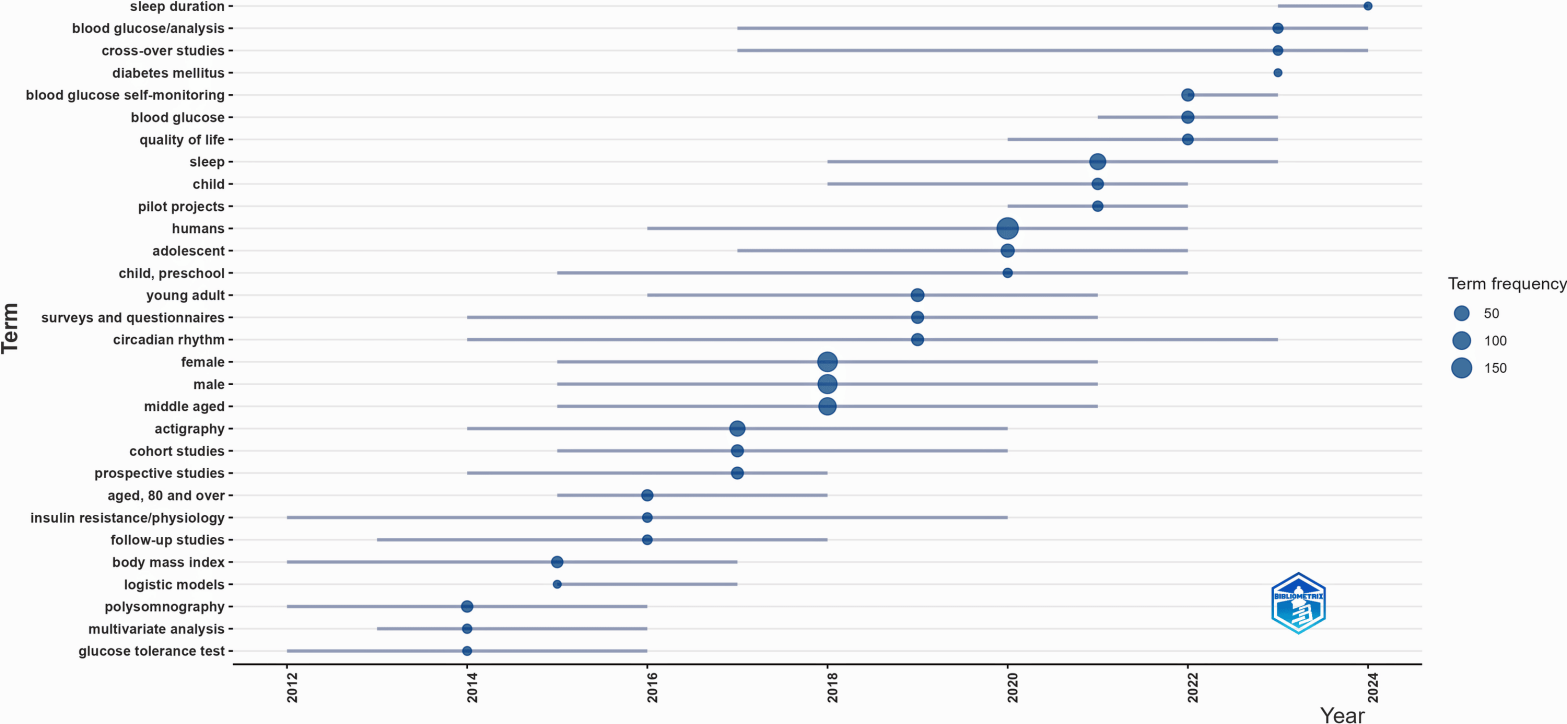
Trending topics in research over time This figure is generated from Biblioshiny.

Figure 6 visualizes the evolution of key research topics over time, with term frequency represented by bubble size. 

Recent years have seen a growing focus on terms such as sleep duration, blood glucose analysis, actigraphy, circadian rhythm, and quality of life. The prominence of blood glucose self-monitoring and diabetes mellitus suggests an increasing emphasis on metabolic health and its relationship with sleep and lifestyle factors. Earlier research trends included PSG, glucose tolerance tests, and insulin resistance/physiology, indicating foundational work in sleep and metabolic disorders. The emergence of child, adolescent, young adult, and middle-aged as keywords points to expanding research across different age groups.

**Figure 7:**
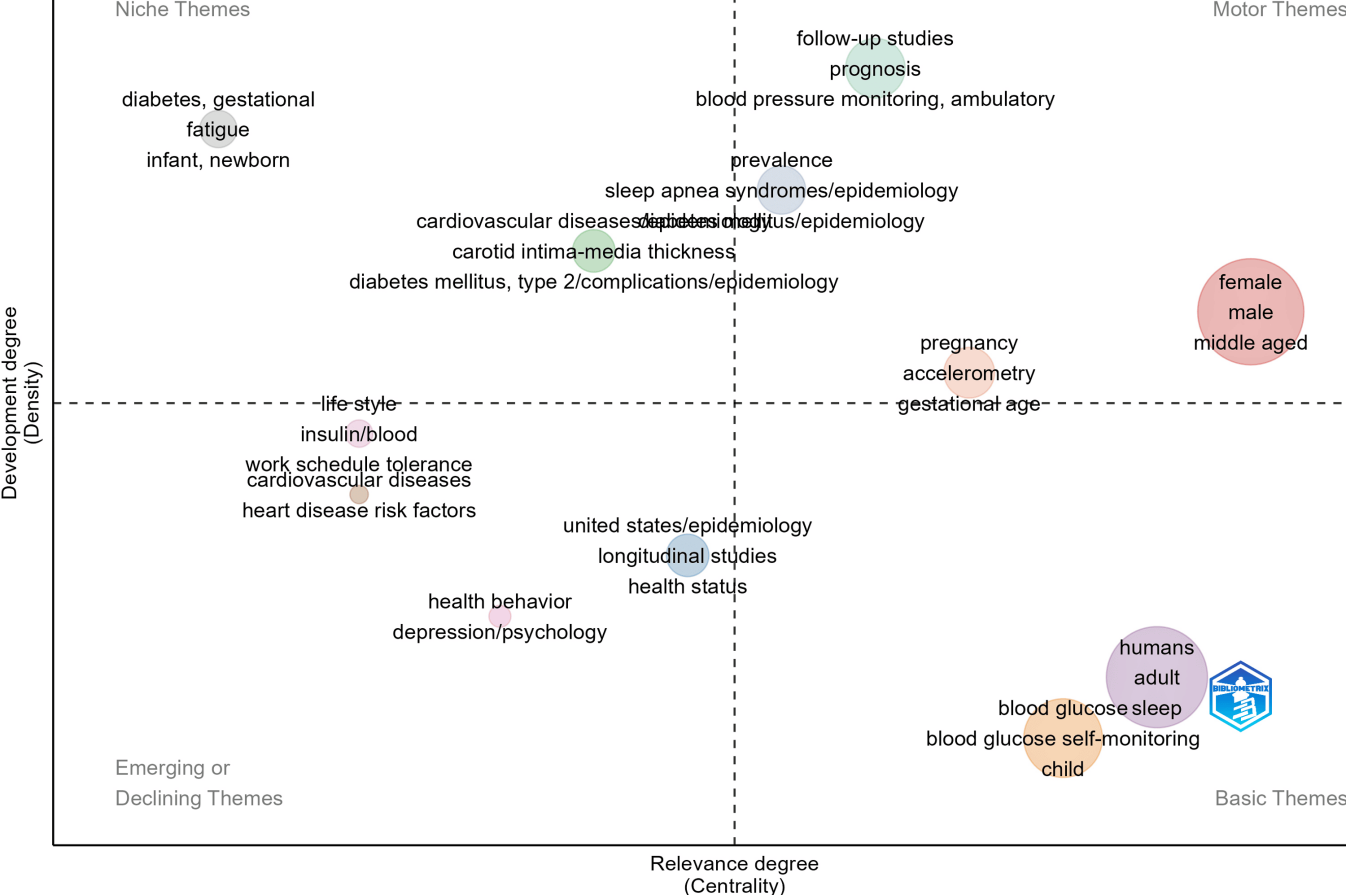
Thematic map of research topics This figure is generated from Biblioshiny.

Figure 7 categorizes research themes based on their centrality (relevance) and density (development stage). 

The motor themes (upper-right quadrant) include female, male, and middle-aged, indicating their well-developed and highly relevant status in the field. The basic themes (lower-right quadrant), such as blood glucose, sleep, blood glucose self-monitoring, and child, represent foundational topics essential to ongoing research. The niche themes (upper-left quadrant), like gestational diabetes, fatigue, and newborn health, are highly specialized but less central to broader discussions. The emerging or declining themes (lower-left quadrant) include health behavior, depression/psychology, and heart disease risk factors, which may be areas of growing interest or losing relevance over time.

Network

**Figure 8:**
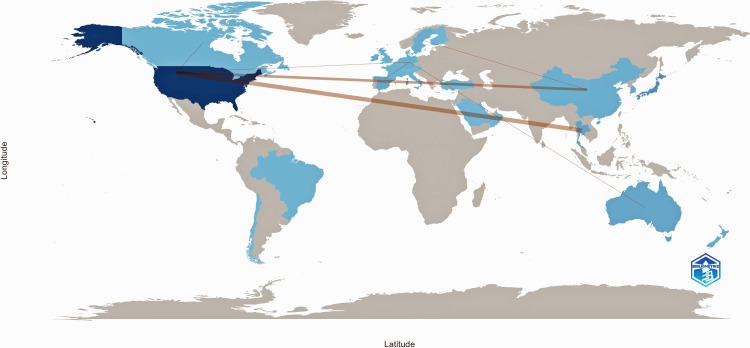
Country collaboration map This figure is generated from Biblioshiny.

This map in Figure 8 visualizes international research collaborations, with lines representing partnerships between countries. 

The intensity of the blue shading indicates the level of research activity, with darker shades representing higher contributions. The United States appears to be the most active collaborator, linking with multiple countries across Europe, Asia, and Australia.

**Figure 9:**
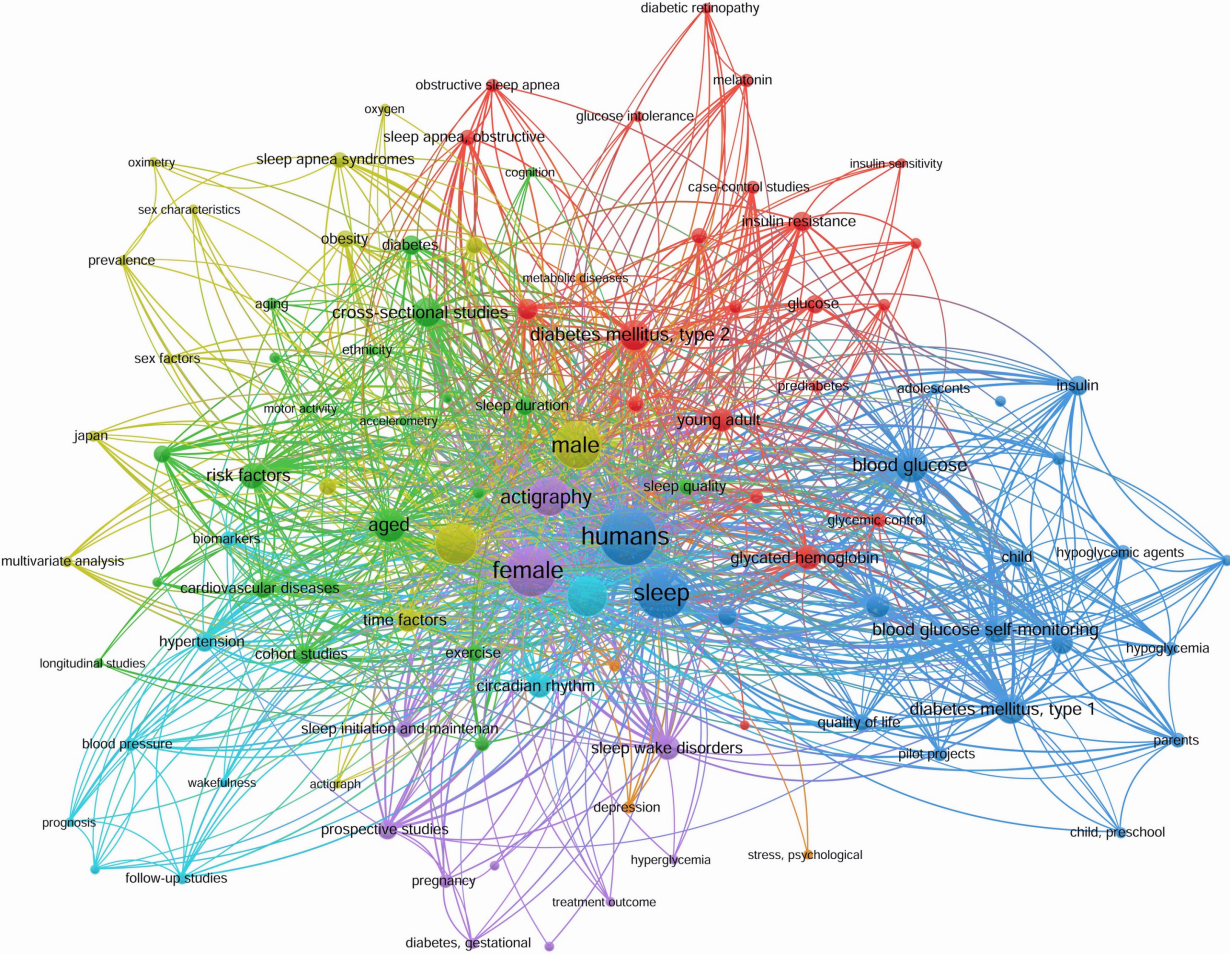
Co-occurrence network of keywords in actigraphy, sleep, and diabetes research This figure is generated from VOSviewer.

The co-occurrence analysis of keywords reveals key research themes in actigraphy, sleep, and diabetes, as shown in Figure 9. 

Major clusters focus on sleep disorders and metabolic health, diabetes and glucose regulation, actigraphy-based objective sleep assessment, cardiovascular and psychological factors, and special populations at risk. These interconnections highlight the growing interest in understanding how sleep patterns influence metabolic and cardiovascular outcomes, particularly in diabetes management.

**Figure 10:**
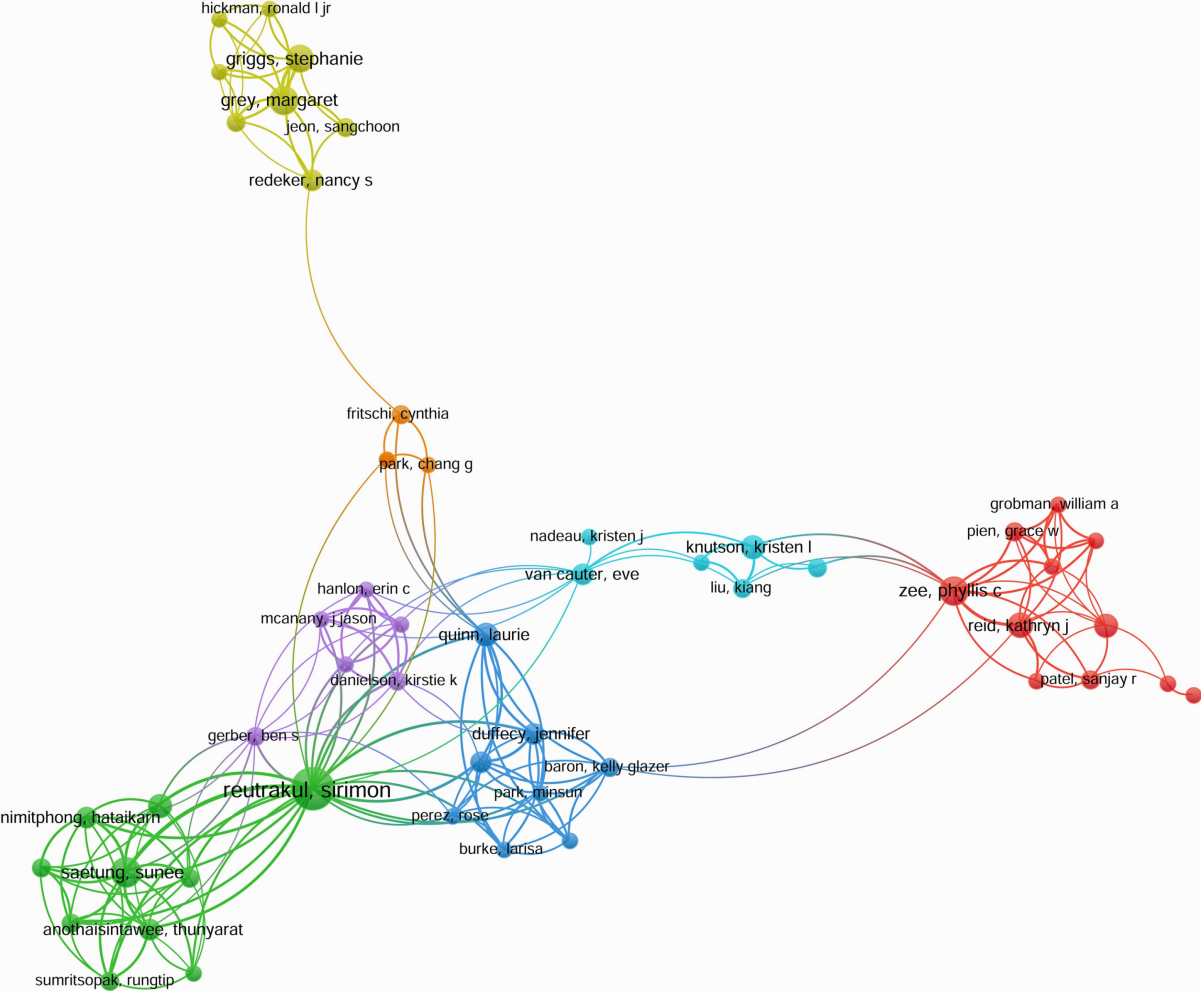
Co-authorship network of research publications This figure is generated from VOSviewer.

The co-authorship network visualizes collaborative relationships among researchers in the domain in Figure 10. 

Each node represents an author, and connections indicate co-authored publications. Stronger connections suggest frequent collaborations, while clusters reveal groups of researchers working closely on related topics. This network highlights key contributors and interdisciplinary linkages in the field.

Discussion

The findings of this bibliometric analysis provide valuable insights into the growing importance of actigraphy in sleep and diabetes research and its implications for both scientific progress and clinical applications. The steady increase in publications and engagement from diverse global institutions suggests that actigraphy is increasingly recognized as a critical tool for understanding metabolic health [15,16]. This trend underscores a paradigm shift in diabetes research, where sleep patterns and circadian rhythms are being acknowledged as modifiable risk factors rather than passive contributors to disease progression [17,18]. The rising interest in this field aligns with broader movements toward personalized medicine and digital health monitoring, where wearable technologies such as actigraphy can enable early detection, intervention, and individualized patient management [19]. 

The dominance of key institutions and core journals in this field highlights the concentration of expertise within specialized research hubs. While this ensures high-quality contributions, it also suggests that collaborations beyond these established centers may be limited. Encouraging research expansion (e.g., targeted funding initiatives, global research networks) in low-resource settings or regions with high diabetes prevalence could improve the generalizability of findings and support the development of context-specific interventions [20]. Additionally, the strong interdisciplinary nature of the research, as reflected in co-authorship networks, signals an ongoing integration of sleep medicine, endocrinology, and metabolic sciences. However, isolated author clusters indicate that knowledge exchange between disciplines could still be enhanced to broaden the scope and application of findings. 

The evolving research themes indicate a shift from basic sleep-disorder characterization to a more nuanced understanding of how sleep influences metabolic processes and quality of life [21,22]. This suggests that researchers are now exploring not just the presence of sleep disturbances in diabetes but also their underlying mechanisms, long-term effects, and potential interventions. The increasing focus on diverse age groups, from children to middle-aged adults, highlights the recognition that sleep-related metabolic dysfunctions develop across the lifespan, reinforcing the need for preventive strategies and early interventions [23-25]. 

The findings also have clinical implications, particularly in integrating objective sleep monitoring into diabetes care. Actigraphy-based studies could help refine guidelines for sleep hygiene, optimize treatment regimens based on circadian profiles, and support behavioral interventions to improve metabolic control [26]. The identification of emerging research areas, such as gestational diabetes, depression, and cardiovascular risk, suggests potential opportunities for targeted research and intervention development [27-29]. 

This bibliometric analysis is novel in its comprehensive evaluation of research trends, thematic evolution, and collaboration networks in the intersection of actigraphy, sleep, and diabetes. Unlike previous reviews that focus on individual studies or specific clinical outcomes, this study provides a macro-level perspective, identifying key contributors, emerging research areas, and interdisciplinary linkages. The integration of co-occurrence and thematic mapping offers insights into how research priorities have shifted over time, highlighting underexplored areas such as gestational diabetes, mental health, and wearable technology applications in metabolic health. However, certain limitations must be acknowledged. The analysis relies solely on PubMed, which, while a robust biomedical database, may exclude relevant studies from engineering, technology, and social science disciplines that also contribute to sleep and diabetes research [30]. Additionally, bibliometric methods assess publication metrics rather than study quality. Finally, while collaboration networks provide valuable insights into research linkages, they may underestimate informal collaborations and interdisciplinary influences that do not result in co-authored publications. Addressing these limitations in future studies by incorporating multiple databases, citation context analysis, and qualitative assessments of research impact would provide a more holistic understanding of the field’s progression.

## Conclusions

This bibliometric analysis highlights the growing significance of actigraphy in sleep and diabetes research, reflecting increasing recognition of sleep as a modifiable factor in metabolic health. Rising publication trends, strong interdisciplinary collaborations, and the concentration of research in key institutions and journals underscore its expanding global relevance. Thematic evolution shows a shift from foundational studies on glucose metabolism to focused investigations on circadian rhythms, self-monitoring, and quality of life. Emerging areas such as gestational diabetes, mental health, and cardiovascular risks offer promising avenues for future research. Greater clinical integration of actigraphy, artificial intelligence-driven analytics, and enhanced global collaboration may advance personalized and adaptive diabetes care.
